# The Escalating Crisis of Health and Safety Law Enforcement in Great Britain: What Does Brexit Mean?

**DOI:** 10.3390/ijerph19053134

**Published:** 2022-03-07

**Authors:** Andrew Moretta, Steve Tombs, David Whyte

**Affiliations:** 1 School of Law and Social Justice, University of Liverpool, Liverpool L69 3BX, UK; andrew.moretta@liverpool.ac.uk; 2 Department of Social Policy and Criminology, The Open University, Milton Keynes MK7 6AA, UK; steve.tombs@open.ac.uk; 3 Department of Law, Queen Mary University of London, London E1 4NS, UK

**Keywords:** occupational safety and health, regulation, self-regulation, UK Government, Brexit, ILO, health and safety enforcement, Health and Safety at Work Act 1974, the Robens Report

## Abstract

This paper explores occupational safety and health regulation in Great Britain following the UK’s exit from the European Union. In particular, the paper focuses on the credibility of regulatory enforcement. The prospects raised by the UK’s exit from the European Union have long been part of a free-market fantasy—even obsession—of right-wing politicians and their ideologues. As the UK’s relationship with the EU is recalibrated, this will present right-wing opportunists with a new rationale for undermining health and safety law and enforcement. The paper uses empirical evidence of Great Britain’s record in health and safety law enforcement to evidence a drift towards an extreme form of self-regulation. It deepens this evidence with a detailed analysis of key international policy debates, arguing that Brexit now raises an imminent threat of the UK entering a ‘race to the bottom’. The paper concludes that the 2021 EU/UK Trade and Co-operation Agreement may enable the UK to evade its formal health and safety responsibilities under the treaty because of the lack of the prospect of significant retaliatory ‘rebalancing’ measures. Should minimal health and safety requirements cease to apply in the post-EU era, then the UK Government will be free to pursue a system of self-regulation that will allow health and safety standards to fall even further behind those of other developed economies.

## 1. Introduction. Self-Regulation and Health and Safety Law in Britain

This article approaches the question of workplace safety regulation from an explicitly critical position. It views regulation as a complex and contradictory process that ultimately seeks to preserve a system of employment in which employers are able to ensure their employees are more likely to absorb the health and safety risks caused by working. In the regulatory balance adopted by most advanced capitalist states, this means an institutionalised tendency towards a system of “self-regulation” [1,2,3].

In Great Britain, this tendency is institutionalised in the work of the regulator, the Health and Safety Executive (HSE). This is the public body that regulates work-related health and safety in partnership with local authorities in accordance with the Health and Safety at Work Act 1974 (HSW Act) through which a variety of existing inspectorates were amalgamated into HSE. The institutional organisation of health and safety regulation in the UK has some anomalies. Whilst the constitutional government authority is the United Kingdom of Great Britain and Northern Ireland, the function of health and safety regulation is split between Great Britain (comprising the nations of England, Scotland and Wales) and Northern Ireland. The statistics referred to throughout the paper generally apply to Great Britain; the sovereign negotiations and representations at an international level are made by the UK government. Separate references to Great Britain and the UK throughout the paper reflect this institutional complexity.

Without entering into detailed discussion of the Health and Safety at Work Act 1974 here, it is worth drawing attention to some of the key features of the *self-regulatory* philosophy which Robens had developed and which were to form the basis of the HSW Act. While the newly formed HSE combined a variety of longstanding regulatory inspectorates, neither this new body nor the existing enforcement agencies were to be considered as any kind of ‘police force’ for industry. According to the Robens philosophy, employers and employees were to co-operatively self-regulate, operating through a variety of formal and informal structures and processes, supported in acting upon their mutual interests by an HSE which would provide advice, encouraging and overseeing compliance [4]. Central to this approach, then, is the use of negotiation and bargaining on the part of regulators to raise, incrementally, general standards of safety management rather than seeking to enforce the meeting of detailed, prescriptive regulations. Further, within this approach, the focus is upon prevention, a future-oriented form of enforcement, rather than punitive modes of regulation, which are cast as backward-looking, seeking to redress past wrongs.

Notwithstanding these commitments, Robens was equally clear that “flagrant offences call for the quick and effective application of the law” and that enforcement should be “rigorous where necessary” [5] (p.80). In other words, even a system of self-regulation ultimately rests upon a range of credible enforcement techniques available to regulators—and these must allow the escalated use of sanctions should the object of regulation fail to co-operate and/or comply with law [6]. This in turn means that even a system of self-regulation requires three conditions to be in place: first, that inspectors actually have a credible presence (to inspect routinely, to investigate incidents) within workplaces; second, that escalation towards greater punitiveness on the part of inspectors is possible and, where the circumstances demand it, likely; and third, that sanctions formally at the disposal of both regulators and thus the courts are credible ones. Yet, if it is questionable whether these conditions have ever in fact been in place since the passage of the HSWAct, then it is certainly the case that, contemporaneously, it is difficult to see any of these conditions of existence of a Robens-style enforcement philosophy in existence [1,7].

Our focus here is on the first two of these three conditions: the presence, or otherwise absence, of a credible enforcement capacity on the part of HSE and local authority inspectors, a matter brought to particular prominence when, in the midst of the COVID-19 pandemic, the UK left the European Union.

This paper uses a primary analysis of three sources of official data to analyse the credibility of the British system of self-regulation. First, it uses official data of enforcement trends, some of which are published by the regulator, and some of which were obtained by requests lodged under the UK freedom of information law (used as the empirical basis for Section 2 and Section 3 below). Second, it develops an analysis of the extensive commentary issued by the European Committee of Social Rights Committee on this issue, and the UK Government’s responses, with a particular focus over the past 4 years. Third, it analyses the published concerns and the direct requests issued by the International Labour Organisation (ILO) Committee of Experts on the Application of Conventions and Recommendations and the UK Government’s responses to those concern and requests.

The paper uses this analysis to build a comprehensive picture of UK compliance with the expectations of international law, based upon the available statistical evidence and on the documented deliberations and debates in international political and legal *fora*.

In the main body of the paper we chart the decline in HSE enforcement capacity in the decade 2010–2020, adding some reflections on such enforcement at the height of the pandemic during 2020–2021, and then, we put this record in the context of Britain’s record of compliance with international standards of enforcement. Finally, the paper sets out what would need to happen to ensure compliance after Brexit.

It should be noted at the outset that our concerns regarding enforcement are focused on the levels of health and safety standards as they exist in British workplaces—there is no claim here that these have or will necessarily translate into recorded rates of injury and ill-health. It has long been recognised that there are significant problems of under-reporting of notifiable injuries and ill-health arising from work in Great Britain. Further, changes in reporting requirements make longitudinal analyses of such rates fraught with difficulty (as do, over longer periods of time, significant shifts in occupational structure as well as in trade union density) [1] (pp. 37–64). However, it *is* the implicit claim here that removing credible law enforcement in this context equates with the removal of protections for workers and members of the public—thus making workplaces more dangerous.

## 2. Towards the End of Enforcement?

The prospects raised by the UK’s exit from the European Union have long been part of a free-market fantasy—even obsession—of right-wing politicians and their ideologues. As the UK’s relationship with the EU is recalibrated, this will present these opportunists on the right with a new rationale for undermining health and safety law and enforcement as an obstacle to economic activity. Yet, at the very same time, longstanding, wholly convincing evidence and arguments, recently spectacularly borne out by the many thousands of excess deaths sustained during the COVID-19 pandemic, have made it objectively apparent that our workplaces require much closer health and safety supervision by an inspectorate able to offer advice and prepared both to issue enforcement notices and prosecute errant employers. Yet, Brexit now raises an imminent threat of the UK entering a highly risky ‘race to the bottom’. As we shall argue, it seems likely that the 2021 EU/UK Trade and Co-operation Agreement (‘TCA’) may enable the UK to evade its *formal* health and safety responsibilities under the treaty because of the lack of the prospect of significant retaliatory ‘rebalancing’ measures. Should minimal health and safety requirements cease to apply in the post-EU era, then the UK Government will be free to permit workplace safety protections to fall even further behind those which characterise other developed economies.

The threat is unquestionably a real and immediate one; even before the TCA had been formally approved by the European Parliament, the UK Government had revealed that it was reviewing employment legislation, starting with the Working Time Regulations- an important element of the EU OHS regime. While the government quickly recanted, there can be little doubt that, despite the UK’s appalling COVID-19 death toll and despite the terms of the TCA, Conservative Party politicians and their ideological allies will seek to build upon previous propaganda successes and seek to persuade the public that rolling back health and safety law and enforcement and extending further the principle of self-regulation is an unequivocal public good.

By way of a preface to an explicit focus on the post-2010 period, it should be emphasised that in May 2004, the Labour Government under Blair, and principally on the initiative of the then Chancellor Gordon Brown, had already formally instituted a programme of ‘Better Regulation’ across all 63 main national regulators and all forms of local authority regulation of business. Health and safety law and regulation was a central target here [2,8]. Attempts through the second half of the first decade of the ‘noughties’ to secure the increasing dominance of Better Regulation proceeded through a series of mutually reinforcing processes, notably: consistent cuts in the budgets allocated to regulators at national and local levels; a long-term rhetorical assault on the very idea of regulation; the establishment of a series of re-regulatory institutions within and of the Government, altering the aims and nature of regulation; various legal reforms which effectively limited regulatory and enforcement capacities and remits; and a constant stream of reviews of regulators and regulation, which both supplied the bases for many of these reforms and initiatives, whilst constantly reminding regulators that their activities and very existence were under constant, critical scrutiny [1,9].

The explicit aim of these initiatives was to further the Better Regulation agenda, an agenda which was based upon a series of unevidenced assumptions. Indeed, as we have shown in previous work, the Better Regulation policy was based upon a set of assumptions that aimed to extend and deepen Robens’ vision of self-regulation [2]. Central here were the following assumptions. First, most companies comply with most regulations most of the time; second, scarce enforcement resources should be targeted at the small percentage of businesses that do not routinely comply; and third, such enforcement activity should largely involve the provision of advice rather than the application of sanctions. Final, underpinning the whole Better Regulation agenda is the belief that *less* regulation and *less* enforcement are the keys to a growth economy [9]. Thus, a combination of political commitments combined with significant resource reductions increasingly required national and local regulators consistently to do ‘more’ with less [10]. The net effect of these pressures was to formalise a system of regulation in the virtual absence of any law enforcement— A political initiative that began in 2004 under a Labour Government but which was to become turbo-charged as, from May 2010, the Conservative-led coalition oversaw austerity and instituted further budgetary cuts across regulatory bodies, alongside a commitment to further undermine business regulation as a shackle on the private corporations which were the engines of any recovery of the British economy. Such ideological, political and fiscal commitments endured through the majority Conservative governments post-2015.

As noted above, HSE inspectors—and their local authority counterparts, health and safety Environmental Health Officers (EHOs)—have *never* been required to act as or seen themselves as a police force for industry, nor really as bodies having or needing to have a significant inspectorial presence. However, Robens’ self-regulatory framework nevertheless required a credible enforcement presence, and that presence has certainly declined over recent years.

This is evidenced by HSE and Local Authority data that we summarise in the following discussion. This data—with the exception of that relating to Covid enforcement—is based upon a combination of publicly available data via the ‘Enforcement’ section of HSE Statistics and ‘Local Authority LAE1 Annual Return Data’ (on enforcement and activity), and a series of Freedom of Information requests made since 2010 (HSE. Freedom of Information Request Reference No: 2010020046, 2014060117, 201608042, 202104190).

This data shows that HSE funding had been reduced significantly: falling from GBP (UK pound sterling) 239 m in 2009–2010 to GBP 121 m in 2019–2020. When inflation-adjusted, this amounts to a real-terms reduction of 58% in central government funding [7]. These funding cuts inevitably affected staffing numbers. If we compare the numbers of HSE full-time-equivalent (FTE) posts, including “Frontline staff” (which includes all inspectors) across the period under examination here, we find that between 2010 and 2020, total HSE staff fell by 36% (from 3702 in 2009/10 to 2371 in 2019/20), with frontline staff, including all inspectors, declining by 28% (1464 in 2009/10 to 1059 in 2019/20). 

Given this decline in funding and personnel, it is hardly surprising that, during this period, every form of enforcement activity declined. The decline in inspectorial presence was particularly stark during this period: between 2010 and 2020, total HSE Field Operations Directorate inspections fell by 72% (from 26,798 in 2009/10 to 7450 in 2019/20).

This low, and declining, number of inspections attests to the declining credibility of the inspectorial presence within workplaces—and is inevitably accompanied by decreases in all forms of formal enforcement activity. Thus we find, for example, that between 2010 and 2020, *total* enforcement notices issued by HSE fell by 27% (from in 9727 in 2009/10 to 7075 in 2019/20) with the most serious, prohibition notices, falling by over 50% (from 3933 in 2009/10 to 1950 notices in 2019/20); meanwhile, there was a total of 885 offences prosecuted by HSE in 2009/2010 leading to 730 convictions, whilst in 2019/20, 517 offences prosecuted by HSE led to 467 convictions in 2019/20—that is, 42% fewer prosecutions and 36% fewer convictions, respectively.

We find similar kinds of trends in the diminution of enforcement capacities and activity in local authorities. For example, between 2010 and 2020:

- The total number of health and safety visits by local authorities fell by 80% (from 196,200 in 2009/10 to 39,200 in 2019/20), of which 6816 were preventative visits, a 94% decline over the decade (there had been 118,000 preventative visits in 2009/10);
- Total enforcement notices issued by local authorities fell by 67% (from 6110 in 2009/10 to 2020 in 2020) with the most serious, prohibition notices, falling by 42% (from 1430 in 2009/10 to 829 in 2020);
- Total offences prosecuted by local authorities fell by 81%, with convictions falling by 78% (there were 284 prosecutions leading to 254 convictions in 2009/2010, while there were 55 prosecutions and convictions in 2019/20, HSE, Freedom of Information Request Reference No: 202104190)

Further, on 1 April 2010, there were 1050 FTE local authority health and safety inspectors; by 31 March 2020, according to those local authorities which recorded this data with HSE, there were just 454, a decline of 57%. Indeed, on 31 March 2020, in some local authority areas there was no bespoke health and safety enforcement regulatory coverage at all: 35 of the 353 local authorities which provided data reported—that is, one in ten—reported having no full-time-equivalent (FTE) health and safety environmental health officer (EHO) in place during 2019/2020; and in total, 129 of the 353 local authorities reported having less than 1.0 FTE health and safety EHO in place during that year (HSE, Freedom of Information Request Reference No: 202104190).

However, this is not just about budgets or personnel or enforcement actions. Two legal reforms in the past decade have made law enforcement either less likely or – incredibly – a breach of administrative law.

The first is the Primary Authority (PA) scheme, which operates at the level of local authority regulation. This scheme, which has been proliferating over the past decade in Great Britain, effectively prevents local authority enforcement actions on health and safety against companies signed on to the scheme. Under PA, a company operating across more than one local authority area enters an agreement with one specific local authority to regulate all of its sites, nationally. This is a legally binding contract which effectively buys for the company the absence of effective oversight in the vast majority of its sites. Enforcement officers across the various local authorities of Great Britain can *visit* the premises of such a company in their own jurisdictions—but they cannot undertake any enforcement activity. Rather, such activity can only proceed through the local authority, which is the PA, and the local authority with which the company has joined a formal, financial contract. The PA scheme is more than a form of regulatory arbitrage which creates inconsistencies or loopholes in law or enforcement to be exploited; rather, it creates formal, robust protection for employers against health and safety law enforcement [11].

Second, many workplaces simply can no longer be inspected proactively *as a matter of law*. In 2011, the UK Department of Work and Pensions (DWP) identified whole sectors of economic activity as ‘low-risk’ and in so doing banned proactive inspections at local authority and then at HSE level. No explanation was ever offered as to how the designation ‘low-risk’ was determined—and one analysis found that of the 258 reported worker fatalities in the 19 months which followed the change, 53% were in so-called low-risk sectors [12].

In short, then, by the time that the precise shape of the UK’s exit from the European Union was in negotiation, health and safety regulation and enforcement was in terminal decline. Meanwhile, the very day of departure, 31 January 2021, was exactly one year after the UK had recorded its first death from coronavirus [13]. By that date, the UK Government’s had recorded 108,764 coronavirus deaths, the highest absolute total in Europe.

In the early period of the pandemic, it was clear that HSE had abrogated its regulatory responsibility for COVID-19 safety. On 27 March 2020, it announced it was suspending all inspections of building sites because it could not guarantee the safety of its inspectors [14]. *Hazards Magazine* meanwhile noted that between March and mid-June, HSE had not conducted one single visit of a care home, despite its responsibility for regulating safety in those sites [15]; by the end of September 2020, it had conducted eight such visits (HSE, *Freedom of Information Request Reference No: 202010347*).

Then, as the Westminster government desperately sought a way out of the first ‘lockdown’, Prime Minister Johnson went on TV on the evening of Sunday 10 May to tell workers in England who had not been going to their normal place of work to return there the next day. Additionally, on that following day, notable for the marked failure of workers to follow the PM’s instruction, Johnson announced in the House of Commons how the Government planned to ensure that workplaces were safe—the government would, he claimed, publish rules for making businesses COVID-19-secure. These would be enforced by the Health and Safety Executive (HSE) through “spot inspections to ensure businesses are keeping employees safe” [16]. He then committed GBP 14 million of additional funding to support the HSE in this task. In fact, a series of *guidelines* rather than legally enforceable rules, accompanied by a drop-in-the-ocean slice of funding in the context of the GBP 100 million plus that the HSE had lost in the previous decade, were clearly never going to be enough to ensure COVID-19-secure workplaces overseen by a protective regulatory agency ready “to police workplace practices as the economy gradually reopens” [17]. Meanwhile, no funds were allocated to local authorities for this illusory regulatory exercise, despite their significant enforcement responsibilities.

It soon transpired that the ‘extra’ funding provided to HSE was to cover “spot inspections”, ostensibly to detect breaches of guidance relating to COVID-19 exposure and transmission. Now, given that HSE is responsible for regulating approximately 5.5 million duty-holders [18], this was a huge task. Additionally, as the data below shows, the GBP 14 million enabled spot checks that reached less than 0.5% of these duty-holders. This response to the pandemic by the UK’s leading safety regulator is revealed as even more problematic when one examines what constitutes a spot check.

As the Lords Minister for the DWP stated in July 2021, “It is important to be clear, however, that these spot-checks are not inspections” [19]. Thus, as HSE had previously noted, what is referred to as ‘spot check calls’ follows a three-stage process, based initially on a telephone conversation “whereby Stage 1 is a scripted question set that follows the COVID-19 guidance, Stage 2 is a more detailed conversation delving into any areas of potential concern from Stage 1” (HSE, Freedom of Information Request Reference No: 202010347). These calls are largely carried out by “approved [privatised] partners to deliver the spot check calls and visits” [20]. In other words, COVID-19 ‘spot checks’ are conducted by telephone calls and largely made by the two private providers to which HSE outsourced the work, namely CDER Group and Engage Services (ESL) Limited (both are ‘debt recovery’ firms).

The use of private outsourcing companies is partly an effect of the rules governing the additional government funding noted above: it cannot be used to train new inspectors. Indeed, more than half of the additional GBP 14 million granted to HSE, GBP 7.2 million, was spent on third-party compliance spot checks (HSE, *Freedom of Information Request Reference No: 20201034*). The Stage 1 scripted calls take outsourced companies 15 min [21]. In May, a YouGov survey of the public reported findings that indicate widespread suspicion of the effectiveness of spot check phone calls. The survey found that 67% of the public supported random in-person HSE checks compared with 9% who thought phone checks would be sufficient [22].

A very small proportion of COVID-19 spot checks led to further action. Some “stage 2 calls are referred on to Stage 3 (on-site inspection) if there are any outstanding concerns from the calls process and it is only at this stage that enforcement action would be taken” [23]. In the six months from 1 April–30 September, a total of 15,622 spot check calls were made, supplemented with 4938 ‘spot check visits’. In total, this COVID-19 enforcement activity generated 78 notices and no prosecutions (HSE, *Freedom of Information Request Reference No: 202010347*).

In addition to COVID-19 checks, in this period, HSE inspectors conducted 406 non-COVID-19 inspections. Taken together, that means that in a six-month period, HSE carried out 5344 inspections or visits to British workplaces— A decline of over 40% from the same six months in the previous year (HSE, *Freedom of Information Request Reference No: 202010347*). As Britain’s workplaces were the site of unprecedented danger, HSE virtually disappeared as an enforcement presence. Indeed, it had become clear that the Robens’s vision of self-regulation had reached its most extreme point in the post-1974 history of UK Health and Safety regulation. Almost a year after Johnson’s assurance that spot checks would keep employees safe, not one single company had been prosecuted for failing to maintain COVID-19-secure workplaces [24]. Meanwhile, by April 2021, over 85,000 Fixed Penalty Notices had been issued against individuals who had allegedly broken COVID-19 guidance [25]. This record unarguably reflects wholesale disregard for the provisions of ILO Convention No. 81 on Labour Inspection that was ratified by the post-war Labour government in 1949. 

This instrument is discussed in section four of this paper, since Convention No. 81 contains the necessary inspection and enforcement standard upon which meaningful implementation of other ILO standards depend. Indeed, implementation of the UK’s major health and safety commitments (notably Conventions Nos. 115, 120, 148) cannot be made meaningful without adequate inspection and enforcement.

## 3. UK Compliance with International Standards after ‘Brexit’

In terms of international law, Brexit should not really have much effect on the UK’s labour protection obligations. Regardless of its relationship with the EU, the UK is, in addition to ILO Convention No.81 on Labour Inspection, bound to comply with both the 1961 European Social Charter and the United Nations International Covenant on Economic, Social and Cultural Rights. These combined obligations arguably require considerably more in terms of workplace health and safety protection that demanded by the *Acquis* [23](p.13). It works like this: The numerous ILO Conventions on occupational safety and health, very few of which have been ratified by the UK, are the primary source of the evolving labour-related standards of both the Charter and the Covenant. The Covenant’s supervisory body, the UN Committee on Economic, Social and Cultural Rights in particular draws very heavily upon ILO instruments and jurisprudence to interpret the Article 7(b) obligation to ensure safe and healthy working conditions. The Committee’s 2016 General Comment on Article 7 cites no less than 17 ‘relevant’ ILO health and safety Conventions to *inter alia* require states to undertake ‘appropriate monitoring and enforcement,’ conduct ‘effective’ investigations and to impose ‘adequate’ penalties on employers in accord with the provisions of those instruments. And not only do a number of EU directives similarly refer to ILO instruments explicitly, as a signatory of the Charter the UK is bound to implement a workplace health and safety regime that is broadly equivalent to that required by the EU *Acquis*. According to the Council of Europe, and to the Charter’s supervisory European Committee of Social Rights, the standards required by EU law closely correspond to those required by the Social Charter, with the ‘rights established by the Charter’ said to be ‘guaranteed in a more or less explicit and detailed manner by EU law’ [26]. Indeed, in relation to the basic Article 3(1) Charter requirement to ‘issue safety and health regulations’, the Committee holds that ‘[a] State Party has satisfied this general requirement if it has transposed most of the *acquis communautaire* on occupational safety and health into its domestic legislation’ [27]. The close relationship between the Charter, EU law and ILO jurisprudences is in turn recognised by the European Commission, which “calls upon all Member States to set an example by ratifying and implementing the ILO conventions classified by ILO as up to date” [28]. Because UN Covenant, European Social Charter and EU requirements and standards are so intertwined with, and draw so heavily upon, ILO health and safety requirements and standards, we can say that the demands of ILO instruments are a benchmark for the standards that international law requires of the UK government. Moreover, that is the position regardless of whether the UK has ratified the ILO instruments in question, and regardless of the extent to which it considers itself to be bound or not bound to the standards EU states must adhere to.

The point is that, whilst the UK government can claim to be no longer bound by EU law, it is still required to maintain an equivalent regime providing at least the same protection. That is the theory. In practice, unfortunately, the UK’s record with regard to employment-related Charter obligations and the obligations of ILO Conventions over the last 40-odd years has been one of failure, non-compliance and disregard for the rule of law. That sorry record indicates that when policy or legislation which will breach labour treaty obligations is contemplated, the hand of Conservative administrations will be stayed only where the reputational and economic disadvantage to the UK is likely to substantially outweigh any perceived political-advantage accruing by pleasing the ideologues, who used to be on the right of the party but now seem to be found in the Conservative Party’s mainstream, and the party’s financial sponsors. As for *ex post facto* findings, no post-1979 Conservative government (including the Coalition Government of 2010–2015) has rectified existing breaches of labour obligations identified by the European Committee of Social Rights or the UN Committee of Economic Social and Cultural Rights, or indeed by the ILO Committee of Experts and the Committee on Freedom of Association. While New Labour administrations occasionally took steps to secure compliance, or at least mitigate the extent of non-compliance, the most egregious breaches relating to freedom of association—restrictions on the right to strike and collective bargaining imposed by the Thatcher and Major governments of the 1980s—were not addressed [29,30] (chapters 5 and 8).

This is in contrast to the grudging respect accorded to decisions of the ECtHR and CJEU. Such are the consequences of failing to eventually, at the very least, appear to comply with the rulings of the Strasbourg and Brussels courts, [31] in terms of state ‘peer pressure’ and loss of prestige, as well as—in the case of the CJEU—significant financial penalties, all UK administrations have invariably ultimately taken steps to secure compliance. While usually preceded by much jingoistic political ‘grandstanding’ by the Conservatives, including various threats to denounce the ECHR and—prior to 2016—calls by Conservative backbenchers for the UK to leave the EU, the Government has always subsequently ‘fallen into line’. However, in the rare instances when UK failures to comply with the standards required by the 1961 European Social Charter or UN Covenant receive publicity and are the subject of Conservative comment, whether from Ministers, backbenchers or media pundits, we tend only to get the jingoism and occasionally muddled or disingenuous claims by the Government that it is either not bound by the instruments in question or by the interpretations of the supervisory bodies. Work and Pensions Secretary Iain Duncan Smith’s ‘fury’ after the European Committee of Social Rights had in Conclusions XX-2 (2013) described certain UK state benefits, including the state pension, as ‘manifestly inadequate.’ The report also indicates that Smith had confused the Charter with the ECHR and had failed to understand how either instrument binds the UK Government [32].

Matters are not helped by the fact that the supervisory mechanisms of the Charter and the Covenant are less than perfect. The overstretched, underfunded, but nevertheless confrontational and strident UN Committee last considered UK compliance with the Covenant in the Concluding Observations on the sixth reporting cycle, published in 2016. The UK state report for the seventh cycle was due to be submitted by 30 June 2021, and we cannot expect the UN Committee’s Concluding Observations on that to be published any earlier than 2023. As for the 1961 Charter, its reporting mechanism works on a four-year cycle, with government reports and the response of the committees referring back to a reference period. Consequently, the European Committee of Social Rights, when considering the report of the UK Government on Article 3 on Occupational Safety and Health in 2021 (a task it last undertook in 2017) will be considering the period 2016–2019. The lack of immediacy aside, the European Committee is largely reliant on the report submitted by the Government, and if any submissions from the trade unions to the UN Committee on health and safety have been put forth, they have yet to be published. Consequently, the UK workplace health and safety regime has been considered only briefly by the UN Committee (or rather its predecessor, the ‘Group of Governmental Experts’) in 1985, and, as will become apparent, the European Committee has yet to get to grips with UK regulatory surrender.

Moreover, as is the case with the ILO—except in instances of the most extreme breaches of the fundamental conventions—the UN Committee of Economic Social and Cultural Rights and the European Committee of Social Rights cannot bring sanctions to bear on errant states. The reporting mechanisms were meant to facilitate the ‘peer supervision’ of state parties which respect the rule of law, and they are ill-suited to dealing with recalcitrant neoliberal states such as the UK. Despite much domestic emphasis on law and order recent administrations appear to believe it to be acceptable to routinely break international law, a tacit policy which manifested itself most recently in relation to the EU/UK Northern Ireland Protocol and the Internal Market Bill [33]. Nevertheless, the pronouncements of the supervisory bodies are authoritative on the question of what their respective instruments require, and thus on whether states are in breach of international law.

Effective enforcement of health and safety regulation is required by Article 3(2) of the European Social Charter. In the Committee’s first interpretative statement on Article 3 it concluded that states must ensure

“regulations are adequately enforced through inspection and civil and criminal sanctions… to safeguard the implementation of the rights to safe and healthy working conditions *in practice*”.

[34]

Moreover, the Committee has consistently held that reports to the committee required by Article 3(2) should document

“the number of visits made by labour inspectorate staff, and the number of enterprises subject to inspection, with a breakdown for the different sectors of activity…number and percentage of workers covered by visits…number of staff employed in labour inspectorates and details of their assignment to the various sectors…this information should be supplied for a period covering the last four years if possible”.

[35]

On the first reading of the report by the UK Government to the European Committee of Social Rights in 2017, one would not have any idea of the long campaign of regulatory surrender set out earlier in this paper. In one passage, the government claims that HSE’s current approach

“accommodates the need for inspectors to target key risks and take proportionate action...inspection is concentrated on the higher risk industrial sectors...However, employers in any sector who under perform in any health and safety may still be visited”.

[36]

The Committee once again still held the UK to be compliant with Article 3(2) the following year [37]. The 1961 procedures, accepting as they are on an uncritical government version of events, have been unable to reveal the UK’s sustained deregulatory campaign. Referring to the UK Government’s report, the Committee noted in 2018

“there have been several government reviews of health and safety…These found no case for radically altering the existing legislation, with the HSE said merely to have ‘revoked and amended legislation to make the legal framework for health and safety clearer by removing unnecessary burdens…”.

[37]

Thus, with a few words, the wholesale emasculation of the system of regulatory enforcement, unarguably in breach of Article 3, can be dismissed as if it were a matter of minor bureaucratic rationalisation. Buried in the Conclusions, however, we can find some sense of unease with the UK Government’s account:

“The Committee notes from the HSE’s Annual Report and Accounts 2014/15 that the number of HSE staff…was 2454 on 31 March 2015 and 3183 on 31 March 2013…The Committee asks that the next report provide detailed information on the new system, particularly with regards to the number of labour inspectors.”

[29,37] (pp. 316–317)

That report was submitted in February 2021, and although the Government would no doubt argue, as it has before, that it has done what the European Committee had asked it to do, it has merely provided the statistics that advice from the TUC led the Committee of Experts to question in their 2019 Observation (see pp. 15–16, below) and chosen not to burden the Committee with any statistics beyond the reference period—those relating to 2019–2020 and 2020–2021 [38].

We will have to wait until the Conclusions of the European Committee of Social Rights are published to see what the Committee makes of the Government’s response. Although members of the ECSR will be aware of the recent dialogue with the ILO Committee of Experts over funding and inspection, and the fact that the statistics it has been presented with are misleading, lacking the input from the TUC enjoyed by the ILO Committee it will have to rely on what the Government chooses to provide. No doubt it will ask for “further and better particulars” as it requested in Conclusions XXI-2 2017 [37], detail which, as we have seen, the Government is required to provide by Article 3(2) of the Charter. Unfortunately, however, even when it eventually does get to grips with the UK’s breaches of the Charter, we can expect little in the way of results. While member states are treaty-bound to act to rectify the failures of compliance the committee identifies, the ultimate sanction—if there is the political will for it—is for a ‘Recommendation’ to be issued by the Committee of Ministers. A small number of recommendations have been issued to the UK. They have, however, largely gone unheeded [29,30] (pp. 120–122, pp. 29–30).

The Charter, ILO and Covenant treaty obligations have been overlaid by the bilateral 2021 EU/UK Trade and Co-operation Agreement (TCA) [39,40]. That treaty explicitly requires the UK to ‘not weaken or reduce’ occupational safety and health protections, and specifically to

“have in place and maintain a system for effective domestic enforcement and, in particular, an effective system of labour inspections in accordance with its international commitments relating to working conditions and the protection of workers…”.

[39] (Chapter Six Article 388)

Very obviously this would appear to compound the C81, Charter and Covenant obligations and arguably include the ILO instruments as yet unratified by the UK but which are drawn upon by the Covenant and the Charter. Those obligations are in turn apparently reinforced by the TCA’s Chapter Eight Article 399(5) requirement that the parties in the agreement, the EU and the UK Government, ‘implement’ the provisions of ratified ILO Conventions and the labour-related Articles and paragraphs of the 1961 European Social Charter [39] (Chapter Eight, Article, 399(5)).

However, the ‘non-regression’ and enforcement clauses are qualified by the obligation only to maintain ‘overall’ standards in the field of occupational safety and health, references to ‘respect for the autonomy of the parties’, which are characteristic of all elements of the agreement, as well as recognition of the right to

“exercise reasonable discretion and to make bona fide decisions regarding the allocation of labour enforcement resources with respect to other labour law determined to have higher priority, provided that the exercise of that discretion, and those decisions, are not inconsistent with its obligations under this Chapter”.

[39] (Chapter Six Article 387 (3))

Moreover, standing to complain of non-compliance is strictly limited to the parties [40] (p.3), and in recent years the Commission has taken a very light-touch approach to monitoring the implementation of EU labour legislation. Member states already enjoyed a considerable margin of appreciation in relation to the methods by which employment rights were enforced, but particularly since the massive expansion of the EU during the first few years of the century the Commission has appeared satisfied merely to see that member states have the workplace health and safety legislation required by the *Acquis* on their statute books [23,29,41]. The days when it was prepared, for example, to challenge the states over matters such as the ‘as far as is reasonably practicable’ qualification which limits the extent of UK employers’ duty of care, arguing that it undermined the protections it was bound to implement as a member of the EU. The Commission unsuccessfully argued that the common law and statutory ‘reasonable practicality’ limitation placed on an employer’s obligation to adopt measures to protect health and safety meant that the UK had failed to meet its obligations under Article 5 of EC Directive 89/391, the famous European Union health and safety “Framework Directive.” [42] have apparently passed, and it is arguably unlikely that it will attempt to enforce what is in any case a standard transnational trade agreement term [41].

As for the prospects of the Commission requiring the UK to ‘implement’ its ILO and Charter obligations, it is unclear whether any departure by the UK from those standards could be said to be a breach of the TCA or whether it is only future departures which may potentially trigger the enforcement mechanisms. Further muddying the waters, a number of EU member states fall short of the standards required by the Charter and by the ILO instruments they have ratified. The consolidating Maritime Labour Convention of 2006 and the Work in Fishing Convention No.188 of 2007 are examples of such collaborations. It also the case that while the EU may implement the provisions of ILO workplace health and safety instruments into European law, the EU cannot itself ratify ILO Conventions. Consequently, while negotiations over EU accession to the Charter are occasionally announced, and the EU has, in close collaboration with the ILO, adopted Directives, the terms of which reflect the requirements of parallel conventions adopted by the International Labour Conference, questions on whether EU states have adequately implemented ILO instruments or the Charter are matters for the individual states and the relevant supervisory bodies, not the European Commission. It might well turn out that these seeming contradictions are indications that Article 399(5) of Chapter Eight, as it now stands, is also merely ‘window dressing’ which will never be enforced [43] (pp. 335-337). That said, the EU–Korea Free Trade Deal of 2011 [44] incorporated a similar clause:

“…The Parties reaffirm the commitment to effectively implementing the ILO Conventions that Korea and the Member States of the European Union have ratified respectively. The Parties will make continued and sustained efforts towards ratifying the fundamental ILO Conventions as well as the other Conventions that are classified as “up-to-date” by the ILO”.

[44] (Article 13.4)

Although Korea had argued that the clause was unenforceable, in the absence of evidence of efforts to implement the fundamental conventions, enforcement action brought by the EU saw the adjudicating ‘Panel of Experts’ finding that it was and that Korea was in breach of its obligations [45], resulting in the notoriously anti trade union state announcing on 4 March 2021 that it was to ratify the fundamental ILO Conventions No. 87 on the right to organise and freedom of association and No.98 on freedom of association and the right to bargain collectively. They were duly ratified on 30 April 2021.

However, the principal drawback to the TCA’s labour protection mechanisms is that they are largely dependent upon it objectively being shown that any perceived dilution of pre-2021 UK labour protection standards impacts significantly upon trade and investment. Despite the pledges to maintain and advance labour protection in the treaty, the TCA essentially allows the UK to diverge from EU labour and workplace health and safety standards if it prepared to risk the imposition of ‘rebalancing’ measures imposed on UK exports to the EU27 [39] (p. 3–4), permitting the UK Government to argue that the TCA does not prohibit Britain from ‘diverging’ from EU standards. Unsurprisingly, given what appears to be the deliberately opaque nature of the labour obligations in the treaty, it is not explicitly stated whether Article 399(5) failures to ‘implement’ ratified Charter provisions or ILO instruments are qualified in this manner. As Ewing has argued, however, ‘although complementary, the obligations in Chapter 8 are not conditional on their impact on trade, but are designed to maintain a basic floor generally’ [43] (p. 311). Grounds for the eventual imposition of rebalancing tariffs or quotas in response to a reduction in the overall level of workplace health and safety protection (including, it must be assumed, working time rights) or failures to enforce domestic protections effectively, are highly qualified and must objectively be shown to impact significantly upon trade and investment [46].

Consequently, even if the European Commission was minded to respond to a dilution of UK workplace health and safety protection, ostensibly in order to protect trade and investment by maintaining the metaphorical level playing field, it would be unlikely to be successful. Even if it were to be successful, the UK need not rectify the situation and subsequent ‘sanctions’ would be strictly limited to proportionate rebalancing measures aimed not at forcing the UK to restore workers’ protections but at restoring the pre-2021 status quo by negating any competitive advantage. There would be no continuing obligation for the UK to restore protections, merely continuing, very likely modest, financial pressure imposed not upon the Government, but upon firms exporting to the EU27.

This predicted reluctance on the part of the Commission to hold the UK to effective enforcement of its labour protection laws and to its ILO and Charter obligations seems likely to be compounded by the fact that it would be unlikely to wish to draw attention to its decidedly relaxed and selective approach to enforcing labour rights. Additionally, as we mourn the passing of the protection conferred by the EU infringement regime upon the labour rights currently enjoyed by UK workers, it is worth emphasizing that in contrast to the vigorous response of the ILO’s Committee of Experts set out in the next section, the Commission failed even to suggest that the UK was in breach of the demands of the EU *Acquis* while UK workplace health and safety policies of ‘regulatory surrender’ were undermining workers’ EU-derived rights to health and safety. To start to call the UK out over regulatory surrender after it has left the EU and to appear to be holding the UK to higher standards than member states would be arguably both be highly questionable and highly unlikely.

Moreover, the EU cannot lay claim to an entirely spotless record where the Charter and ILO Conventions are concerned: The CJEU has handed down rulings at variance with the fundamental ILO Conventions 87 and 98 on freedom of association and the right to organise and bargain collectively, and with Article 6(4) of the European Social Charter, which requires states to guarantee workers the freedom to take industrial action—as well as arguably Article 28 of the EU Charter of Fundamental Rights on the ‘Right of collective bargaining and action.’ Perhaps worse still, the Commission along with the IMF and the ECB required states seeking financial assistance from the ‘Troika’ to withdraw sectoral collective bargaining systems, an initiative at odds with C98, with Article 6 of the Charter, as well as arguably Article 12(1) of the EU Charter [47]. Neither that shameful episode, nor the CJEU’s highly unsatisfactory *Viking* [48] and *Laval* [49] line of cases which essentially rank business rights ahead of the rights of workers to strike and bargain collectively in the European jurisprudential order, are easily reconcilable with the values and principles of the EU, and are certainly at odds with those of the ILO and Council of Europe as embodied in the fundamental ILO Conventions and the Charter. It seems unlikely that the Commission will wish to revisit these matters when it stands to gain so little. In short, despite the platitudes offered by the Commission, it is highly unlikely that the post-Brexit settlement will provide any bulwark against the current UK government’s self-regulatory extremism.

## 4. Compliance with International Standards: The ILO and C81

Article 16 of Convention No. 81 stipulates that ‘Workplaces shall be inspected as often and as thoroughly as is necessary to ensure the effective application of the relevant legal provisions’; Article 17(1) provides that “[p]ersons who violate or neglect to observe legal provisions enforceable by labour inspectors shall be liable to prompt legal proceedings without previous warning…”; while Article 18 requires that “adequate penalties for violations of the legal provisions enforceable by labour inspectors… shall be provided for by national laws or regulations and effectively enforced.” Yet, as we have noted, in Great Britain prosecution and enforcement action has declined markedly over the past 2 decades.

It is not only the scale of coverage and the reasonable chances of violators being prosecuted that concerns Convention 81. The coverage must be consistent and leave no workplaces outside the scope of regulators. Thus, Article 2(1) stipulates that:

“The system of labour inspection in industrial workplaces shall apply to all workplaces in respect of which legal provisions relating to conditions of work and the protection of workers while engaged in their work are enforceable by labour inspectors”.

Yet, from 2011 onwards the Tory–Liberal Democrat Coalition government in the UK created a new category of ‘low risk’ workplaces, which in effect exempted the majority of UK workplaces from unannounced ‘proactive’ inspection. Some of the most obviously deadly sectors were relieved of any realistic prospect of receiving HSE or local authority attention, with the magazine *Hazards* subsequently finding that 53% of recorded workplace deaths had occurred as a result of injuries sustained in the course of what were now deemed ‘low risk’ working activities [11].

Moreover, Article 10 of Convention 81 provides that: “The number of labour inspectors shall be sufficient to secure the effective discharge of the duties of the inspectorate.” By ILO benchmarks the UK should have a ratio of inspectors to workers of at least 1: 10,000. The current ratio in the UK is roughly 1:15,000. In order to comply with Convention 81, HSE and local authorities would need to increase the number of their inspectors by at least 50%.

The UK’s programme of underfunding regulators and the consequent reduction in regulatory activity has had an obvious and inevitable impact and a deleterious effect on the numbers of prosecutions and other enforcement actions. It is very difficult to plausibly argue that there exists in the UK a credible enforcement regime, and there can be no doubt that the UK falls well short of compliance with the standards demanded by international law.

The ILO’s Committee of Experts on the Application of Conventions and Recommendations (CEACR) has, with the assistance of the TUC, for some years taken a close interest in the incompatibility of the UK’s policies of ‘regulatory surrender’ with the C81 treaty obligations. Unfortunately, it is reliant on pointing out to the Government its obligations—the demands of international law—and the extent to which the UK regime falls short of those requirements and, with the help of the TUC, coaxing it towards compliance. Nevertheless, within the strictures of the ILO constitution, the Committee harries the Government politely but mercilessly.

In the early 1990s, as the first European Community (as it then was) Directives explicitly concerned with workplace safety began to be implemented, a spate of ‘Observations’ followed three ‘Direct Requests’ adopted after concerns had been raised by the TUC about reductions in workplace inspections. In a fourth Direct Request, adopted in 1995, information was requested following a report by the TUC that the UK was not compliant with its C81 obligations. This was a time when UK employers were able to anticipate only *one inspection in every 7 years*, and it appears to have been at this point that the Committee became officially conscious that the Government was effectively emasculating the legislation it was obliged to implement. In its characteristic diplomatic manner, it told the Government that it:

“regrets the reluctance on the part of HSE to enforce legislation arising from EC (EU) Health and Safety Directives. According to the TUC unless an employer knows that prosecution is likely in cases of health and safety offences, large sections of industry will fail to adopt a positive approach…”.

[50])

Three more Direct Requests were adopted in 2000, 2003 and 2009 following on from Observations made in the same year. However, only in the Direct Requests of 1993 and 1995 did the Committee make its dissatisfaction entirely clear, arguably a reflection of the more benign approach to workplace health and safety funding, and to inspection, taken during the first two New Labour administrations. Similarly, out of six adopted between 2000 and 2016, only the 2013 and 2016 Observations were overtly critical of the UK’s policy of regulatory degradation, responses to the overt attacks on HSE and local authority funding overseen by the Liberal Democrat–Conservative Coalition. The 2013 Observation was adopted after the CEACR had officially been advised of the cuts to workplace inspection which had followed the 2011 ‘Good Health and Safety, Good for Everyone’ programme, implementing the plan to cut inspections in ‘non major hazard’ areas by one-third each year referred to above. Concerned that no less than 10 Articles of the Convention might have been breached, information was requested on the criteria used to targeting inspections, including on whether the relevant trade unions and employers’ organisations were consulted on the selection of workplaces for inspection; on whether self-assessment in low-risk sectors was voluntary or mandatory; and the extent to which the inspectorate was able to identify employers guilty of ‘underperformance’ operating in these sectors. Detailed information was also requested on the impact of these measures and that of the HSE’s then-new Fees For Intervention scheme (‘FFI’ are charges levied against errant employers ‘to pay for the time it takes to identify the breach and put things right’) on the numbers of accidents and the incidence of occupational disease [51].

The response of the Government was disappointing, and the result was that the 2016 Observation was—at least by the unfailingly diplomatic standards of the CEACR—couched in more confrontational language. The Committee requested more from the Government, and the post-2011 withdrawal of inspection in supposedly low-hazard sectors was revisited:

“workplaces identified with lower safety risks do not necessarily have a lower incidence of cases of occupational disease, and that they should therefore not be categorised as low risk”.

[52]

The Committee considered “it important that certain, often vulnerable, categories of workers...are not excluded from protection” as a consequence of this targeting [52], and it now wanted more information than it had required in 2013. It requested statistics on inspections undertaken, particularly inspections of SMEs; detail on the numbers of infringements detected; and information on the numbers of inspectors and on budget allocations since 2011. It now wanted more information on the targeting criteria; the Committee ‘also once again’ requested “that the Government provide information on the means used by the labour inspectorate to detect underperformance in the area of workplace health and safety of workplaces that are currently not expected to be subject to inspections” [52]. It required reassurance that workplace whistle blowers be assured confidentiality in their dealings with the HSE and ‘once again’ requested:

“that the Government provide information on whether the use of self assessments in workplaces not subject to inspection was voluntary or mandatory, asking the Government to indicate whether self inspection data ‘are fed into the inspection programme process and to indicate that all workplaces remain liable to inspection by the labour inspectorate”.

[52]

It also asked for more detail on the FFI scheme, on the extent of HSE reliance on the money generated and requested information

“on any measures by the Government to avoid potential damage to the reputation of the HSE concerning impartiality and independence”.

[53]

In 2019, having received fresh information from the TUC on the continuing failure of the Government to respond to the concerns voiced by the Committee, it now chose to explicitly refer in an Observation to the

“substantial budgetary cuts imposed…within the last ten years leading to an insufficient number of labour inspectors (including technical experts) to effectively enforce compliance with the labour legislation (for example, in the areas of asbestos and fire safety) and that this number is further declining”.

[52]

Turning to statistical information provided by the Government, the Committee noted with concern

“that the number of labour inspectors decreased from 1432 to 990 between 2011–2012 and 2018–2019. In this respect, it notes the indication of the TUC that the actual number of labour inspectors is significantly lower, as managers and technical experts are included in the Government’s figures”.

[53]

Stepping the pressure up, the Committee recalled,

“that the number of labour inspectors shall be sufficient to secure the effective discharge of their duties, the Committee requests the Government to take the necessary measures in this regard, and to provide information on the measures it is taking with respect to recruitment and retention. It requests the Government to continue to provide statistics on the number of labour inspectors, and to provide its comments with respect to the indication of the TUC on issues arising from the current classification of technical experts and managers as inspectors”.

[53]

The Committee noted that according to the UK Government “the number of workplace health and safety inspections undertaken between 2011 and 2012 and 2018 and 2019 remained relatively stable with around 20,000 visits a year, and only slightly decreased by about 2000.” Aware that visits are not the same things as inspections, it requested “the Government to continue to provide information on the number of labour inspections undertaken, and to provide its comments as regards the observations made by the TUC” [54]. In particular it requested detail on the intelligence use to target workplaces for inspection.

Drawing on the HSE 2018–2019 report, the Committee returned “with concern” to the cuts in HSE funding and increased reliance on FFI as well as the potential threat to the reputation of the HSE for impartiality presented by FFI and the possibility that employers may be disinclined to seek help and advice for fear of being targeted for revenue purposes:

“The Committee recalls that, in conformity with Article 11, it is essential for member States to allocate the necessary material resources so that labour inspectors can carry out their duties effectively. The Committee emphasizes that labour inspection is a vital public function, at the core of promoting and enforcing decent working conditions. It urges the Government to take the necessary measures to ensure that sufficient budgetary resources are allocated for labour inspection, and to provide information on the concrete steps taken to address the challenges identified by the HSE with respect to budgeting. In this respect, the Committee requests the Government to continue to provide information on the budgetary situation of the HSE, and the proportion of its budget raised from the FFI scheme…”.

[53]

Turning its attention to Articles 17 and 18 on enforcement the Committee noted that the HSE’s 2018–2019 report states that between 2015 and 2016 and 2018 and 2019 the number of cases that led to a conviction or verdict fell from 672 convictions (out of 711 cases with a verdict) in 2015–2016 to 361 convictions (out of 396 cases with a verdict) in 2018–2019, and that:

“the HSE is currently examining the reasons for this decrease… In this respect, the Committee also notes the reference of the TUC to a substantial decline in the enforcement activities of the labour inspection services mainly due to a decline in the staff number and inspections. The Committee requests the Government to take the necessary measures to ensure that adequate penalties for violations of the legal provisions enforceable by labour inspectors are effectively enforced, in conformity with Article 18 of the Convention. In this respect, it requests the Government to provide information on the outcome of the assessment regarding the reasons for the above-mentioned decrease. It also requests the Government to provide detailed information on the number of infringements detected and the measures taken as a result, disaggregated by workplaces covered in small, medium-sized and large enterprises and the sectors concerned”.

[53]

Having in October 2020 received observations from the TUC communicated along with a new Government report and a subsequent response to the TUC from the Government, the Committee recalled that the Government had claimed the “effectiveness of the HSE demonstrates that there are sufficient numbers of inspectors to secure the effective discharge of its duties.” Rather than explicitly call that claim out as absurd, the Committee cited the HSE’s 2019–2020 annual report for “indicating that there were 1059 inspectors, visiting officers and regulatory compliance officers in the HSE as of March 2020, compared to 1066 in March 2019”, adding that the TUC had revealed “that there are only 290 full-time equivalent main grade regulatory inspectors for the whole of the United Kingdom, excluding Northern Ireland” [54].

The ‘additional funding’ issued in 2020 boasted of by the Government was similarly disparaged, with the CEACR stating that

“according to the TUC, the additional staff secured using this financing can only be hired under fixed-term contracts, for this financial year. The TUC also indicates that the additional resources have been used to offer HSE staff overtime”.

[54]

The Committee once again reinforced the message that the Government was in breach of C81, albeit couched in characteristic conciliatory language:

“Reaffirming that labour inspection is a vital public function, at the core of promoting and enforcing decent working conditions, and recognizing the particular challenges faced by the country in the COVID-19-19 context, the Committee requests the Government to continue to take the necessary measures to ensure that sufficient budgetary resources are allocated for labour inspection”.

[54]

Returning to Articles 17 and 18 and enforcement, the Committee pressed harder for information on why the number of prosecutions had decreased:

“… according to the Annual Report of the HSE 2019–2020, there were 355 prosecution cases… as compared to 396 in 2018–2019 and 509 in 2017–2018.’ Acknowledging that increased penalties were making cases more complex and expensive and involving more of the inspectorate’s time, the Committee has now requested that ‘the Government to continue to provide information on its assessment of the factors impinging on the HSE’s prosecution work and on any measures taken or envisaged to improve the situation, including increased allocations for inspector time and resources, and to provide information on any consultations [with the TUC] held on this matter. The Committee also requests the Government to provide further information on the role of inspectors in the process, including the time and resources dedicated to these legal proceedings as a proportion of overall inspector time and resources”.

[54]

Obviously, there is much here for UK activists and for Parliament to work with, and it is unfortunate that the excellent work of the CEARC is not afforded more publicity. However, a progressive UK Government could very easily formally link the shortfalls of the British workplace health and safety regime identified by the Committee with select committee scrutiny. Indeed, it could arguably produce political capital by instituting a programme of ratifications of up-to-date workplace-health-and-safety-related instruments of ILO Conventions and Protocols and Recommendations in general. Such an initiative augmented by fresh constitutional mechanisms entrenching the ILO instruments and requiring the Government and the courts to take into account the work of the Committee along the lines of the Human Rights Act 1998 would do more than alleviate the post-Brexit vulnerability to overt workplace health and safety deregulation and renewed covert regulatory surrender. In particular, if a similar approach was taken to the obligations imposed by the labour- and employment-related Articles of the 1961 European Social Charter accepted by the UK, it would effectively require genuine dynamic alignment with EU standards regardless of the European Commission’s over-relaxed attitude towards requiring EU states to comply with the EU workplace health and safety regime.

## 5. Conclusions

This is the state of affairs that faces the UK at the outset of the post-Brexit regulatory settlement. While it is difficult to see how workplace health and safety regulatory standards could fall any further, as we indicated in the introduction to this article, there is very obviously some expectation in government and among Brexit fundamentalists that there will be some kind of deregulatory ‘windfall’ on the cards. Workplace health and safety legislation of EU origin has long been a totemic symbol of European interference to the Conservative party in the UK. The Working Time Directives, although drained of as much substance as possible when implemented and further emasculated by the familiar tactic of non-enforcement [29] (pp. 327–341), are especially loathed.

Even now that the COVID-19 crisis has shown the UK’s workplace health and safety enforcement regime to be “a hollowed out shell” and to have killed thousands of British workers as a result [7], it would be unrealistic to expect that the Government, guided as it is by ideology rather than pragmatism, will not wish to continue down the road of regulatory surrender and extremist self-regulation.

The TCA, for all its faults, has potential. First, as we have seen, the TCA requires that the UK maintains “a system for effective domestic enforcement and, in particular, an effective system of labour inspections in accordance with its international commitments relating to working conditions and the protection of workers” [39] (Chapter Six Article 388), an obligation which, if enforced with sufficient vigour, would be of tremendous value in focusing the Government’s attention on the necessity to comply with the rule of law on the international plane and rejuvenate British workplace health and safety. Moreover, in relation to ensuring current protections are kept in place, the TCA ostensibly requires the UK not to weaken or reduce its domestic regulatory regime. A failure to resolve disputes over health and safety provisions could result in the imposition of trade tariffs. This provision could, if it were ever to be triggered, enact some considerable economic damage [41]. Nevertheless, as we have seen, these enforcement mechanisms are not all they seem. This potential aside, however, the TCA as it now stands offers little incentive to impose a compliant health and safety regulatory regime, or credible political disadvantage to the government should it withdraw or dilute employment rights [40,43,46].

To make the skeleton commitments to “system for effective domestic enforcement” set out in the TCA more meaningful, the agreement would need to be amended to include the following. First, in addition to tariffs and quotas, dissuasive financial penalties should be incurred in the event that workplace health and safety and labour standards diverge to any significant extent *in practice*, arguably making genuine mutual dynamic alignment between the UK and the theoretical demands of the *acquis*, rather than the very approximate alignment that pertains among the EU27, much more likely. Second, the link between the enforcement regime and trade and investment should be loosened, the concept of proportionate ‘rebalancing measures’ replaced by explicitly punitive measures and the dilution of labour rights unequivocally prohibited. Third, the link between the TCA and respect for the ILO and Charter jurisprudences should be strengthened with a mutual programme of ILO workplace health and safety and working time instrument ratifications initiated with the EU undertaking to implement the provisions of the ILO instruments into European law, the UK undertaking to ratify or adopt the relevant instruments and EU accession to the European Social Charter.

That, however, is very much the ideal, a ‘wish list’, dependent not only on the inclination and political judgement of a future progressive UK Government but on the appetite of the European Commission and 27 EU member states for binding the Acquis more tightly to the Charter and ILO jurisprudences. With some of the member states now being led by administrations considerably further to the right of the political spectrum than our own Government, the sharpness of that appetite is questionable.

Irrespective of the TCA, and of the politics of the EU, in the UK there are ways to address the self-regulatory extremism that has shattered the credibility of workplace health and safety enforcement in the UK. The government could ratify the Revised Social Charter of 1996. That contains a refreshed Article 3 of the 1961 Charter, fleshed out to reflect the 1967–1996 ECSR jurisprudence, as well as a new Article 21 on information and consultation and a new Article 22 guaranteeing workers the “right to take part in the determination and improvement of the working conditions and working environment”. Article 22(1)(b) refers specifically to occupational health and safety. Article 21 and 22 are Articles 2 and 3 of the Additional Protocol of 1988 which predates the Revised Charter. It must nevertheless be borne in mind that the mere fact of ‘ratification’ tells us little about the implementation of a convention or protocol, nor about the extent to which the terms of an instrument are enforced. More significantly, the UK could reinvigorate the moribund monitoring and enforcement of our Charter obligations and follow the example of Finland by ratifying the Collective Complaints Protocol to permit the TUC to present complaints directly to the European Committee of Social Rights. The ILO rights and freedoms and the Charter rights relating to labour and employment could be entrenched in the UK constitution in the manner of the European Convention of Human Rights under the terms of the Human Rights Act 1998 with courts obliged to take account of the findings of the relevant supervisory committees.

Again, it must be acknowledged that in the current political climate, those proposals may plausibly be described as little more than wishful thinking. However, given that that those measures would be intended to deliver the minimum standards that the TCA purports to currently hold the UK to, they could scarcely be said to be radical proposals.

Yet, in the context of the UK’s appalling record in observing ILO workplace health and safety instruments they may appear radical. Maritime instruments aside, the UK government ratified 32 ILO conventions in the years spanning 1945–1980. Just two of those conventions addressed workplace health and safety standards. During 1980–1997, the years of the Thatcher and Major governments, other than three maritime conventions and two conventions, the ratification of which was initiated by the Callaghan government (including the important but partially ratified workplace health and safety instrument, the Working Environment (Air Pollution, Noise and Vibration) Convention 148 of 1977), only the uncontroversial Labour Statistics Convention was ratified.

In total, the UK government has ratified only 6 of a total of 35 ILO ‘up-to-date’ health and safety protocols and conventions. This is the same number as the following nations: Cameroon, Comoros Islands, El Salvador, Guyana, Libya, Lithuania, Mauritius, Mozambique, North Macedonia and Saudi Arabia. Indeed, there are a total of 74 countries that have ratified more ILO health and safety conventions than the UK. This is scarcely what should be expected of a supposedly world leading economy currently engaged in negotiating trade deals with the world beyond the EU 27.

It does not appear anything is going to change on this front. The current UK Labour Party, in opposition, does not appear to have the determination to organise around such an agenda. Political commitments to workplace health and safety in the wake of Brexit and the COVID-19 crisis are few and far between with the 2019 Labour Party election manifesto commitment to establishing a Royal Commission apparently now having been dropped. Had Jeremy Corbyn been elected as Prime Minister in 2017, it is possible that we would have seen a new Committee considering the 21st Century world of work 50 years after Robens was appointed by the Labour government Secretary of State for Employment, Barbara Castle.

This leads us to a familiar point: the real outcomes of workplace regulation do not depend on the ebb and flow of politics, whichever direction it flows in. Regulatory outcomes may be improved or made worse by international political settlements, but those outcomes ultimately rest on the capacity of workers to resist and organise in their workplaces to ensure that health and safety standards are upheld.
